# Poor Competitiveness of *Bradyrhizobium* in Pigeon Pea Root Colonization in Indian Soils

**DOI:** 10.1128/mBio.00423-21

**Published:** 2021-07-06

**Authors:** Danteswari Chalasani, Anirban Basu, Sarma V. S. R. N. Pullabhotla, Beatriz Jorrin, Andrew L. Neal, Philip S. Poole, Appa Rao Podile, Andrzej Tkacz

**Affiliations:** a Department of Plant Sciences, School of Life Sciences, University of Hyderabad, Hyderabad, Telangana, India; b Department of Plant Sciences, University of Oxford, Oxford, United Kingdom; c Department of Sustainable Agriculture Sciences, Rothamsted Research, North Wyke, United Kingdom; University of Nebraska-Lincoln

**Keywords:** pigeon pea, *Bradyrhizobium*, competition, bacterial community, 16S rRNA gene amplicon

## Abstract

Pigeon pea, a legume crop native to India, is the primary source of protein for more than a billion people in developing countries. The plant can form symbioses with N_2_-fixing bacteria; however, reports of poor crop nodulation in agricultural soils abound. We report here a study of the bacterial community associated with pigeon pea, with a special focus on the symbiont population in different soils and vegetative and non-vegetative plant growth. Location with respect to the plant roots was determined to be the main factor controlling the bacterial community, followed by developmental stage and soil type. Plant genotype plays only a minor role. Pigeon pea roots have a reduced microbial diversity compared to the surrounding soil and select for *Proteobacteria*, especially for *Rhizobium* spp., during vegetative growth. While *Bradyrhizobium*, a native symbiont of pigeon pea, can be found associating with roots, its presence is dependent on plant variety and soil conditions. A combination of 16S rRNA gene amplicon survey, strain isolation, and co-inoculation with nodule-forming *Bradyrhizobium* spp. and non-N_2_-fixing *Rhizobium* spp. demonstrated that the latter is a much more successful colonizer of pigeon pea roots. Poor nodulation of pigeon pea in Indian soils may be caused by a poor *Bradyrhizobium* competitiveness against non-nodulating root colonizers such as *Rhizobium*. Hence, inoculant strain selection of symbionts for pigeon pea should be based not only on their nitrogen fixation potential but, more importantly, on their competitiveness in agricultural soils.

## INTRODUCTION

Pigeon pea (*Cajanus cajan* [L.] Millspaugh) is one of the most important legume crops, with diverse uses as food, feed, fodder, and fuel, besides enriching soil through biological nitrogen fixation. Globally, the crop is grown on about 7 million hectares (1), mainly as a rain-fed crop in semiarid tropical and subtropical regions of South Asia, East Africa, Latin America, and the Caribbean. It is the primary source of dietary protein for over a billion people in developing countries. Millions of resource-poor smallholder farmers grow this multipurpose crop with minimal inputs to sustain their livelihoods. Domestication of the wild progenitor species Cajanus cajanifolius (endemic to the Indian subcontinent) resulted in the origin of the cultivated pigeon pea in central India more than 3,500 years ago, from where it subsequently spread to other parts of the globe (2, 3). India is the largest producer of pigeon pea, accounting for 72% of global production (1). It is the second-largest cultivated legume crop (after chickpea) in India, contributing 15% by area and 17% by production (4). The major pigeon pea growing zones in India can be divided into the south zone (Andhra Pradesh, Telangana, and Karnataka), central zone (Madhya Pradesh, Maharashtra, and Gujarat) and northern plain zone (Uttar Pradesh) (5). The states of Andhra Pradesh, Madhya Pradesh, and Uttar Pradesh record the highest yields (6). The soil types in these three states located in the south, central, and northern zones are red soil (alfisol), black soil (vertisol) and alluvial soil (inceptisol), respectively, based on the U.S. Department of Agriculture Soil Taxonomy (Table S1). A large number of pigeon pea varieties are cultivated in India, exhibiting a vast genetic and phenotypic diversity of agro-morphological traits, including variations in plant type, branching pattern, pod and seed size, seed color, protein content, grain yield, resistance/tolerance to abiotic and biotic stresses, crop duration, photoperiod sensitivity, and days to flowering and maturity (7).

10.1128/mBio.00423-21.10 TABLE S1 Soil type and genotype data, sample factors, Bash code, data used for Fig. S4, S5, S6, S7, and S8 construction, ANOSIM output, SIMPER output, information regarding isolates from root and soil, and information regarding WGS strains. Download Table S1, XLSX file, 0.7 MB. Copyright © 2021 Chalasani et al. 2021 Chalasani et al. https://creativecommons.org/licenses/by/4.0/ This content is distributed under the terms of the Creative Commons Attribution 4.0 International license.

10.1128/mBio.00423-21.4 FIG S4 (A to D) Microbial community structure visualized with PCoA plots at different taxonomic levels; (A) zOTU level, (B) genus level, (C) family level, and (D) phyla level of Indian pigeon pea and British Arabidopsis thaliana, bread wheat, Medicago truncatula, and Pisum sativum. (E) PCA plot with rhizoplane and root endosphere samples showing six dominant phyla abundances shaping the community (Alpha-, Beta-, and Gamma- stand for *Proteobacteria* classes). (F) Same as panel E with *Rhizobium* and *Bradyrhizobium* species removed *in silico*. For PCoA plots, *n *= 713; for the PCA plot, *n *= 303. Download FIG S4, PDF file, 0.4 MB. Copyright © 2021 Chalasani et al. 2021 Chalasani et al. https://creativecommons.org/licenses/by/4.0/ This content is distributed under the terms of the Creative Commons Attribution 4.0 International license.

10.1128/mBio.00423-21.5 FIG S5 Shannon entropy associated with each fraction (indicated by color) and presented for each soil; from left, alfisol, inceptisol, and vertisol and each genotype inside the soil cluster; from left to right, Asha, Durga, and MKK. The outcome of the *t*-test for initial soil against each fraction with Bonferroni correction is indicated above the bar plots; *, *P* < 0.05; ***, *P* < 0.001. Download FIG S5, PDF file, 0.02 MB. Copyright © 2021 Chalasani et al. 2021 Chalasani et al. https://creativecommons.org/licenses/by/4.0/ This content is distributed under the terms of the Creative Commons Attribution 4.0 International license.

10.1128/mBio.00423-21.6 FIG S6 Pigeon pea microbiota taxonomic profile at the phylum, family, and genus levels for each developmental stage, fraction, soil, and genotype. Soils abbreviated as A, alfisol; I, inceptisol; V, vertisol and each genotype inside the soil cluster; from left to right, Asha, Durga, and MKK. Download FIG S6, PDF file, 0.1 MB. Copyright © 2021 Chalasani et al. 2021 Chalasani et al. https://creativecommons.org/licenses/by/4.0/ This content is distributed under the terms of the Creative Commons Attribution 4.0 International license.

10.1128/mBio.00423-21.7 FIG S7 Volcano plots for the microbial community during the plant’s vegetative stage. Each genus is presented as a dot, where the *x* axis indicates the fold difference between the bulk soil and the respective fraction (in logarithmic scale) and the *y* axis indicates the significance according to the *t* test *P* value corrected with the false-discovery rate and presented as –log scale [–log(0.05 = 1.301 and presented as a dotted gray line on the plots]. Selected genera are annotated. Download FIG S7, PDF file, 0.4 MB. Copyright © 2021 Chalasani et al. 2021 Chalasani et al. https://creativecommons.org/licenses/by/4.0/ This content is distributed under the terms of the Creative Commons Attribution 4.0 International license.

10.1128/mBio.00423-21.8 FIG S8 Volcano plots for the microbial community during the plant’s flowering stage. Each genus is presented as a dot, where the *x* axis indicates the fold difference between the initial and the respective fraction (in logarithmic scale) and the *y* axis indicates the significance according to the *t* test *P* value corrected with the false-discovery rate and presented as –log scale [–log(0.05) = 1.301 and presented as a dotted gray line on the plots]. Selected genera are annotated. Download FIG S8, PDF file, 0.4 MB. Copyright © 2021 Chalasani et al. 2021 Chalasani et al. https://creativecommons.org/licenses/by/4.0/ This content is distributed under the terms of the Creative Commons Attribution 4.0 International license.

The root-associated bacterial communities of many plants and crop species have been extensively studied, including those associating with other N_2_-fixing legumes, such as soybean, alfalfa, red clover, common bean, and Lotus japonicum (8–14). However, the bacterial community of tropical grain legumes such as pigeon pea has not been described yet. The root bacterial communities of legumes differ from those of non-legumes owing to the symbiotic association with diverse rhizobia in the root nodules. The legume hosts exert a strong influence on the rhizobial diversity patterns in the soil and different parts of the root bacterial community (15).

Studies based on strain isolation from India suggest that pigeon pea can be nodulated by *Rhizobium* spp. (16–18), *Bradyrhizobium* spp. (19), *Sinorhizobium/Ensifer* (20–22), *Mesorhizobium* (18, 22), or even *Burkholderia* (18). However, in other geographical regions, including Cote d’Ivoire (23), Ethiopia (24), Dominican Republic (25), and Brazil (26), pigeon pea is nodulated solely either by *Bradyrhizobium* spp. or *Sinorhizobium*/*Ensifer* (27), suggesting that any other species found in the nodulation studies may need to be reevaluated.

Apart from rhizobial symbionts, pigeon pea harbors diverse non-rhizobial root colonizers belonging to *Bacillus*, *Brevibacillus*, *Paenibacillus*, *Lactobacillus*, Pseudomonas, *Agrobacterium*, Enterobacter, Klebsiella, *Chryseobacterium*, *Streptomyces*, *Serratia*, and other genera (16, 20, 23, 28).

Most studies have concentrated on isolation and characterization of pigeon pea nodule and root bacteria and their use in inoculation assays to promote plant growth. No comprehensive study on the root-associated bacterial community of pigeon pea or any other legumes grown in the Indian soil has been undertaken to date, nor has any throughput screening of common pigeon pea symbionts been performed. Genomic tools and high-throughput sequencing technologies allowed characterization of the genetic and genomic diversity of pigeon pea, including whole-genome sequencing (3), although this did not cover the root bacterial community. The present study was designed to (i) identify bacterial taxa associated with pigeon pea roots and surrounding soil, (ii) investigate the factors shaping the pigeon pea root-associated bacterial community in various Indian soils, and (iii) identify nodule symbionts.

Pigeon pea is a legume and is able to obtain nitrogen through symbiosis. However, its growth is often supplemented with inorganic and organic fertilizers, as there are reports of weak nodulation in some parts of India (29). Some varieties of pigeon pea have a low symbiosis potential, and it is possible to obtain nodulation-deficient lines by simply crossing less efficient lines between each other (30).

We hypothesize that the reported low nodulation efficiency of pigeon pea is an outcome of either the low number of compatible symbionts in the soils and/or their low competitiveness in colonizing the host plant.

To capture the representative bacterial community of pigeon pea, we have sampled different parts of the plant microbiome (root endosphere, rhizoplane, rhizosphere, and soil not attached to the roots—loose soil, representing only a very weak plant influence). All the plants in our assay were nodulated. We did not separate nodules from the roots and sequence them but, rather, collected them in a separate experiment for isolation study. Based on our previous experience (31), we know that nodule bacterial community structure, obtained through next-generation sequencing is likely to include data for bacteria attached to the outside of the nodules and/or those able to (re)colonize the nodule, especially in its later developmental stages.

To separate the effect of actively growing roots secreting photosynthetic products into the surrounding soil from generally weaker plant secretions (32) and those potentially serving only as an attachment point for microbes (33), we have sampled plants at two contrasting developmental stages, vegetative and flowering.

The widely grown pigeon pea cultivars Asha (ICPL 87119), Durga (ICPL 84031), and Mannem Konda Kandi [MKK] (ICPH 2740) were grown in three soils collected across the Indian subcontinent, representing three major geological substrates for soil formation—alfisols (red soils originating from highly weathered rocks, typical of the tropical climate process of leaching most soil minerals accumulating insoluble aluminum and iron), inceptisols (alluvial soils originating from recent flood deposits), and vertisols (black soils originating from older fluvial deposits and representing a typical clay-rich tropical agriculture soil) (34).

We compared the Indian pigeon pea data with our previously characterized legume and non-legume plant bacterial community of British soils. The data are fully comparable, as the same methods were applied for all the samples.

We show in the 16S rRNA gene amplicon screen, bacterial isolation assay, and a gnotobiotic experiment that pigeon pea roots are predominantly colonized by non-symbiotic *Rhizobium* spp., rather than symbiotic *Bradyrhizobium* spp.

## RESULTS

After quality control and demultiplexing, we obtained 5.1 million reads in total with an average of 11,443 reads per sample. After *in silico* mitochondrial and protoplast contamination removal, we standardized the samples to 1,000 reads each, removing 3% of samples that did not reach that sequencing depth.

### Fraction, developmental stage, soil, and genotype shape the pigeon pea bacterial community.

To understand what is shaping the pigeon pea bacterial community, we used multifactor permutational multivariate analysis of variance (PERMANOVA) of the following factors: plant fraction (root endosphere, rhizoplane, rhizosphere, and loose soil), soil type (alfisol, inceptisol, and vertisol), plant genotype (Asha, MKK, and Durga), and plant developmental stage (vegetative and flowering) as factors.

**FIG 1:**
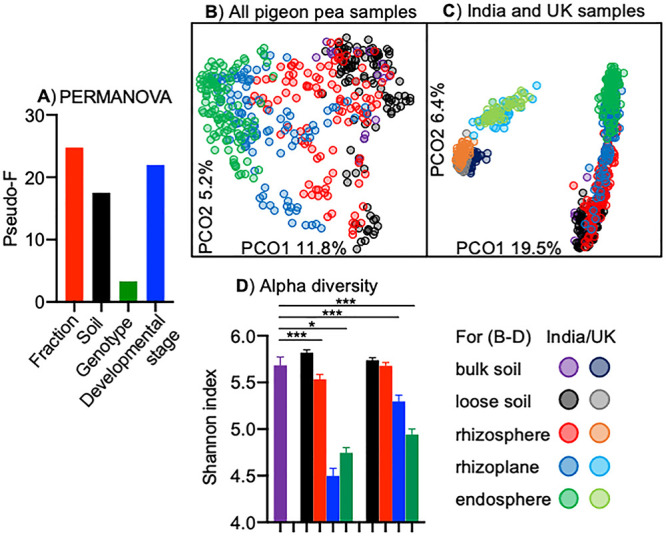
Community structure and diversity of pigeon pea-associated microbiota. (A) Influence of different factors on microbiota using the PERMANOVA pseudo-*F* value as a proxy; (B) PCoA plot representing pigeon pea microbiota and shown with the visual separation fractions; (C) PCoA plot representing pigeon pea microbiota and British soil-grown Arabidopsis thaliana, wheat, Medicago truncatula, and pea; (D) Shannon entropy shown for each fraction. The outcome of a *t* test for bulk soil against each fraction with Bonferroni correction is indicated above the bar plots; *, *P* < 0.05; ***, *P* < 0.001. For panels A, B, and D, *n *= 449, while for panel C, *n *= 713.

We found that the main factor controlling the assembly of the pigeon pea bacterial community is the plant fraction, followed by developmental stage, and soil type, and the least important, yet still a significant factor, is the plant genotype (Fig. 1A and Fig. S1). However, when we look at each fraction separately, the soil is more important than the developmental stage for loose and attached soil, while the developmental stage is the dominant factor for rhizoplane and endosphere (Fig. S1B). Comparing each soil separately, fraction is more important than developmental stage for alfisol, and they are of approximately the same importance for inceptisol, while developmental stage is more important for vertisol (Fig. S1C). All factors exert a similar influence for individual plant genotypes (Fig. S1D). Analyzing the data with the separation for vegetative and flowering stages, we uncovered that while fraction and plant genotype are of similar importance for both these stages, the soil type factor is more important for the flowering plants (Fig. S1E).

10.1128/mBio.00423-21.1 FIG S1 PERMANOVA output measuring the influence of factors on microbiota using the pseudo-*F* value as a proxy. (A) All pigeon pea data. (B) Pigeon pea data split by each fraction. (C) Pigeon pea data split by soil. (D) Pigeon pea data split by genotype. (E) Pigeon pea data split by developmental stage. (F) A comparison of Indian pigeon pea samples and British plants at different prokaryotic taxonomic units. Download FIG S1, PDF file, 0.03 MB. Copyright © 2021 Chalasani et al. 2021 Chalasani et al. https://creativecommons.org/licenses/by/4.0/ This content is distributed under the terms of the Creative Commons Attribution 4.0 International license.

We have also compared the major factors of pigeon pea bacterial community assembly with our previous findings using legume and non-legume plants grown in the United Kingdom (31). Even the strongest factor as fraction is dwarfed by the importance of the sample origin (India versus United Kingdom). Some of this difference can be explained by the plant species’ influence. However, distantly related plants grown in the United Kingdom (pea, *Medicago*, wheat, and *Arabidopsis*) have a relatively similar bacterial community compared to the Indian-grown pigeon pea, suggesting that the plant species effect is small.

At the zero-radius operational taxonomic unit (zOTU) level, the origin is approximately 10-fold more influential than fraction, while this ratio decreases with increasing taxonomic level (6× for genus, 5× for family, and 3× for phylum). This change is probably caused by a reduction of alpha diversity with an increase in taxonomic level.

Visualizing the bacterial community using principal-coordinate analysis (PCoA), we confirmed PERMANOVA results where fraction (Fig. 1B and Fig. S2A) and then developmental stage (Fig. S2D), soil (Fig. S2B), and plant genotype (Fig. S2C) can shape the pigeon pea bacterial community. PCoA plots demonstrate that plants of all three genotypes shift their bacterial community between vegetative and flowering stages, an effect especially observed for vertisol-grown plants (Fig. S2B and D). This observation is a plausible mechanism behind the increase of soil type factor strength for flowering plants already reported using PERMANOVA (Fig. S1E).

10.1128/mBio.00423-21.2 FIG S2 (A to D) PCoA plots representing pigeon pea microbiota and shown with the visual separation by (A) fractions, (B) soil type, (C) plant genotype, and (D) plant developmental stage. *n *= 449. Download FIG S2, PDF file, 0.2 MB. Copyright © 2021 Chalasani et al. 2021 Chalasani et al. https://creativecommons.org/licenses/by/4.0/ This content is distributed under the terms of the Creative Commons Attribution 4.0 International license.

Analysis of similarity (ANOSIM) and PCoA plots were used to assess the differences between samples based on specific factors. For the fraction factor, the major community shift happens between rhizosphere and rhizoplane (ANOSIM *R* = 0.301, *P* < 0.01), followed by rhizoplane and endosphere (*R* = 0.269, *P* < 0.01), while there is less difference between loose soil and rhizosphere (*R* = 0.176, *P* < 0.01) or bulk soil and loose soil (*R* = 0.096, not significant). The PCoA plot clearly illustrates it with a gentle sample location shift between all fractions, while the main boundary can be drawn between the bulk soil-loose soil-rhizosphere cluster and the rhizoplane-endosphere cluster (Fig. S2A).

For the soil type, ANOSIM separates plants grown in vertisol from ones grown in alfisol (*R* = 0.289, *P* < 0.01) and inceptisol (*R* = 0.287, *P* < 0.01), while the difference between alfisol- and inceptisol-grown plants is weaker, yet significant (*R* = 0.199, *P* < 0.01) (Fig. S2B). Plant genotype is a significant factor shaping the bacterial community (based on PERMANOVA [Fig. 1A]) when the influence of all the three genotypes is considered. However, ANOSIM reveals that no genotype-to-genotype comparison is significant (Asha-Durga *R* = 0.028, Asha-MKK *R* = 0.018, and Durga-MKK *R* = 0.018; *P* > 0.05 for all). PCoA plot visualizes the lack of separation for samples based on their plant genotype factor (Fig. S2C). ANOSIM confirms PERMANOVA findings that developmental stage is one of the strongest factors (*R* = 0.211, *P* < 0.01), while PCoA plot clearly separates samples based on their developmental stage, and as expected, loose soil is not affected by the plant developmental stage (a cluster of red-labeled points in the top right corner of Fig. S2D).

To confirm the observed PCoA sample-spread pattern and the main factors driving the community assembly, we reanalyzed the data originally based on zOTU assignment (sequencing read similarity) at the three higher taxonomical levels of genera, family, and phyla (Fig. S3). Irrespective of the taxonomic level, the fraction, followed by developmental stage, soil type, and genotype, are the main factors controlling the community structure. Not surprisingly, the higher taxonomic levels, due to the reduced number of categories, have lower alpha diversity, leading to a better separation of different sets of samples. This effect can be observed with an increased PCoA axis (PCO 1 and PCO 2) contribution in explaining the data variation (i.e., PCO 1 axis for genus level explains 18.9%, for family 24%, and for phyla 43.9%). Moreover, the PERMANOVA pseudo-*F* value also increases for the higher taxonomic level data separation (apart from soil type at the phylum level).

10.1128/mBio.00423-21.3 FIG S3 PCoA plots representing pigeon pea microbial community structure at the phyla, family, and genus taxonomical levels using fraction, soil type, genotype, and developmental stage. The same colors are used as in Fig. S4. For each panel, the number in the bottom right indicates the PERMANOVA pseudo-*F* value for a given factor sample separation. All comparisons, *P* < 0.01. *n *= 449 samples. Download FIG S3, PDF file, 0.5 MB. Copyright © 2021 Chalasani et al. 2021 Chalasani et al. https://creativecommons.org/licenses/by/4.0/ This content is distributed under the terms of the Creative Commons Attribution 4.0 International license.

To analyze the Indian pigeon pea bacterial community in a wider context, we have supplemented the data with our previous legume and non-legume soil and root assay of plants grown in the United Kingdom (31). For consistency, we analyzed the data at four taxonomic levels—zOTU, genus, family, and phylum. However, irrespective of the taxonomic level used, we see that PCoA plots clearly separate the Indian from the United Kingdom samples on their first axis (PCO 1), while the fractions within each origin group are separated on the second axis (PCO 2) (Fig. 1C and Fig. S4A to D). The fraction separation pattern is similar for both Indian and United Kingdom samples. Root samples (endosphere and rhizoplane) in both cases are separated from the soil fractions (loose soil and rhizosphere), and with a higher taxonomic level, root bacterial community becomes similar across both geographical locations, irrespective of the plant species or genotype origin.

This community convergence was analyzed further with PCA, and both the United Kingdom and Indian root communities are highly influenced by *Alphaproteobacteria* and *Bacteroidetes* (Fig. S4E). As we analyze legume plants, we present the data with *in silico* removal of potential symbionts, such as *Rhizobium* and *Bradyrhizobium.* However, such removal does not change the main PCA-based sample separation and overall pattern (Fig. S4F). Pigeon pea roots at the flowering stage (with or without potential symbionts removed) when the rhizodeposition may be reduced have enriched their bacterial community to *Gammaproteobacteria* and *Actinobacteria*. As we have sampled only vegetative stage plants in United Kingdom samples, we cannot confirm here whether this process is uniform or specific only to the Indian pigeon pea samples.

There is a significant reduction of alpha diversity expressed as Shannon entropy associated with the rhizoplane and endosphere of pigeon pea, irrespective of their developmental stage. However, alpha diversity is higher during the flowering rather than vegetative stage. There is no consistent soil or genotype influence on alpha diversity (Fig. 1D and Fig. S5).

### Pigeon pea roots are colonized by *Proteobacteria* and *Bacteroidetes*.

**FIG 2:**
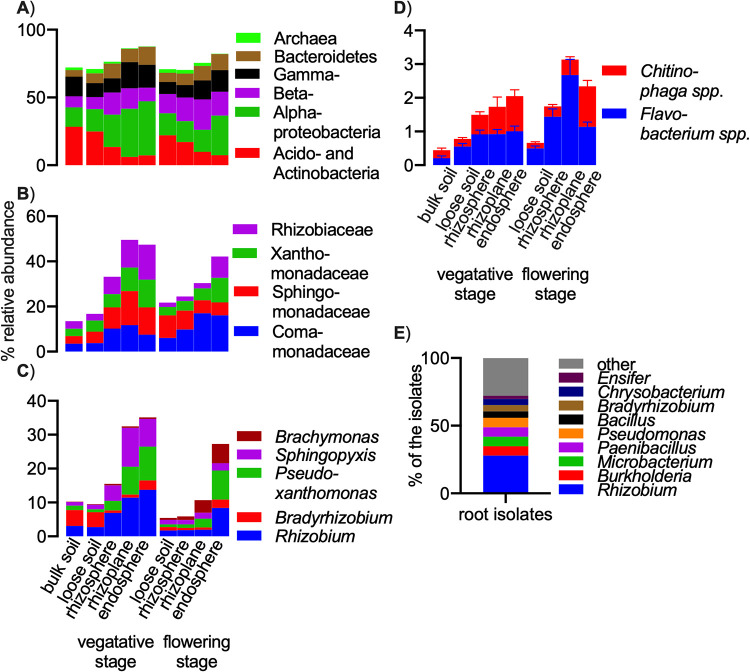
(A to C) Taxonomic profile of pigeon pea microbiota at the (A) phylum, (B) family, and (C) genus levels. The top seven phyla, top four families, and top five genera are shown as a percentages of the total community. (D) potential beneficial genera belonging to the *Bacteroidetes* phylum. (E) Taxonomic profile of the bacterial isolates from roots of pigeon pea grown in native soils.

Taxonomic profiles of the rhizoplane and root endosphere are different from those of the loose soil and rhizosphere. Root fractions are colonized by *Alpha*-, *Beta*-, and *Gammaproteobacteria*, as well as *Bacteroidetes*, especially during vegetative growth. The *Proteobacteria* replace *Acidobacteria*, *Actinobacteria*, and Archaea found in soil (Fig. 2A and Fig. S6).

*Comamonadaceae*, *Sphingomonadaceae*, and *Xanthomonadaceae* abundance increases in the rhizosphere and root fractions, while *Rhizobiaceae* are more prevalent in the roots of vegetative plants. The separation of soil and root fractions is more apparent during vegetative growth than at the flowering stage (Fig. 2B and Fig. S6).

The main genera in the roots of vegetative stage plants are *Rhizobium*, *Pseudoxanthomonas*, and *Sphingopyxis*. Some plant roots also have a high abundance of *Bradyrhizobium*, suggesting an active endosymbiosis. Vegetative plants allow *Sphingopyxis* root colonization, which is replaced by *Brachymonas* in the flowering stage (Fig. 2C and Fig. S6). Similarity percentage analysis (SIMPER) run on the endosphere samples from vegetative and flowering stages places these two genera as the most influential taxa for the community separation between these developmental stages (Table S1).

As *Bacteroidetes* (along with *Proteobacteria*) increases its abundance in the root fraction, we analyzed this phylum in more details. *Bacteroidetes*, and especially *Chitinophaga* spp. and *Flavobacterium* spp., were found to reduce pathogen load inside the plant roots of sugar beet with target antibiotic production by overexpressing polyketide synthases and nonribosomal peptide synthetase genes (35). In our study, we found these two *Bacteroidetes* genera to be more abundant in the roots than in the rhizosphere or soil, with their abundance being especially high in older plants (Fig. 2D).

We investigated genus abundance in more detail using volcano plots. Here, we present the increase and decrease in bacterial community abundance with statistical power. For clarity, we compared plant selection in each soil type at the vegetative and flowering stages, where each genus is represented by a dot of a different size according to its total abundance and is located on the *x* axis according to its abundance in a given fraction against the bulk soil control. The *y* axis indicates the statistical confidence for suppression (if on the left-hand side of the graph) or selection (if on the righthand side of the graph) (Fig. S7 and S8).

The loose soil community becomes different from the bulk soil over time; while there are almost no genera either suppressed or selected in the vegetative stage, they do appear during the flowering time, signifying at least some plant roots’ influence over the bacterial community thriving in the more distant soil. For both plant developmental stages, the rhizosphere is a place of suppression of *Bradyrhizobium* and *Rhizobium* in alfisol and *Bacillus* in vertisol. For the vegetative stage, rhizoplane and endosphere selection are clear, especially for *Rhizobium*, *Pseudoxanthomonas*, and *Sphingopyxis* (genera belonging to the *Alphaproteobacteria*). There is soil type specificity in the suppression/selection pattern; plants grown in alfisol suppress *Bradyrhizobium*, and those grown in vertisol suppress *Bacillus*, while in inceptisol, plants strongly select for *Rhizobium*. In general, plant roots exert a weaker influence in their flowering comparing with the vegetative stage, while *Hydrogenophaga*, *Sphingomonas*, *Opitutus*, and *Brachymonas* replace *Rhizobium*, *Pseudoxanthomonas*, and *Sphingopyxis* as efficient root colonizers (Fig. S7 and S8).

### High rate of *Rhizobium* in pigeon pea roots.

**FIG 3:**
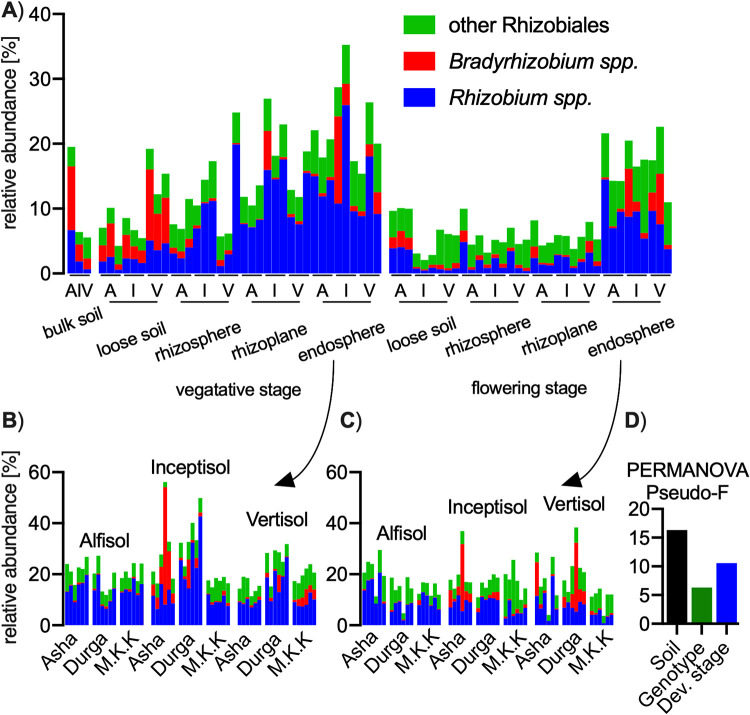
(A to C) *Rhizobium* spp., *Bradyrhizobium* spp., and other *Rhizobiales* community taxonomic 16S gene amplicon profiles associated with pigeon pea plants (A) for all fractions, (B) for vegetative stage endosphere showing all biological replicates, and (C) for flowering stage endosphere showing all biological replicates. (D) PERMANOVA output for the relative strength of *Rhizobiales* on the endosphere community with the separation for the soil type, plant genotype, and developmental stage influence (*P* < 0.001 for all comparisons). Soils abbreviated as A, alfisol; I, inceptisol; V, vertisol.

During sampling, we kept the nodules attached to the roots. We therefore expected to observe a spike of nodule symbiont abundance in the root samples. Bulk soil and loose soil contain relatively high proportions of *Bradyrhizobium*, while pigeon pea rhizosphere and roots are colonized predominantly by *Rhizobium* (Fig. 3A). The proportion of *Rhizobium* is reduced in the flowering stage (average [av.], 3.5%) relative to the vegetative one (av., 8.7%), while the abundance of *Bradyrhizobium* and other *Rhizobiales* is relatively stable, with only *Bradyrhizobium* reduction in loose soil over the plant lifetime. Focusing on plant roots only, we observed both the soil type and plant genotype specificity in *Rhizobiales* selection (Fig. 3B and C). In general, plants (all the biological replicates for a given condition) are either highly colonized by *Bradyrhizobium* (as Asha in inceptisol) or by *Rhizobium* (Durga in inceptisol and vertisol). The soil type influence on the *Rhizobiales* community inside plant roots is comparable to the general soil type influence (PERMANOVA pseudo-*F *= 17.5 for all bacterial community taxa and 16.3 for *Rhizobiales* only); the importance of genotype increases almost 2-fold (3.4 to 6.3 for the all-bacterial community and *Rhizobiales* community, respectively), while developmental stage, still being important, has a reduced pseudo-*F* value from, 21.9 to 10.6 (Fig. 3D and Fig. 1A). This signifies that *Rhizobiales* spp. are more influential than other bacterial community taxa inside legume plant roots, but their community stays relatively stable over the plant lifetime.

In order to explain the higher abundance of *Rhizobium* over the pigeon pea native symbiont *Bradyrhizobium* (and *Ensifer*), we isolated bacteria from the soil and roots of pigeon pea grown in Indian soils.

We isolated and purified 60 colonies from the rhizosphere and 272 colonies from the root endosphere and nodules, of which 13 and 43, respectively, were found to be unique strains. Isolates of root-inhabiting *Rhizobium* contribute to 28% of the abundance, followed by *Burkholderia*, *Microbacterium*, *Paenibacillus*, and Pseudomonas, with 7% each. *Bradyrhizobium* isolates make up 5%, while *Ensifer* represents only 2% of the isolated community (Table S1). These values are broadly consistent with the root 16S gene amplicon screen output.

We sequenced the genomes of *Rhizobium/Agrobacterium* (11 isolates), *Burkholderia* and *Paraburkholderia* (4 isolates) and *Microbacterium* (3 isolates); none were associated with any *nod* or *nif* genes, suggesting these strains are not symbionts. We also sequenced many *Bradyrhizobium* nodule isolates and confirmed their nodulation ability. However, a detailed discussion of nodule-isolated strains will be presented in a separate publication.

We confirmed that *Bradyrhizobium* rather than *Rhizobium* nodulates pigeon pea by growing plants in controlled conditions of growth chambers in Oxford, UK, in sterile vermiculite. Plants inoculated with *Rhizobium* isolates (either single or in a mixed inoculation) were not nodulated, while their roots contained a high bacterial presence of ∼10^7^ CFU per root. Plants inoculated with *Bradyrhizobium* (either single or in a mixed inoculation) were nodulated (2 to 5 nodules per plant) and contained a similar bacterial presence to plants inoculated with *Rhizobium* isolates only. Plants inoculated with both *Rhizobium* and *Bradyrhizobium* strains (strain mixtures) formed nodules, but only *Rhizobium* was recovered from the roots (rhizoplane and endosphere combined), while all the visible nodules harbored *Bradyrhizobium* strains only.

## DISCUSSION

We identified that the principal factor controlling the assembly of the pigeon pea bacterial community was the plant fraction, followed by developmental stage, and soil type; the least important, but a significant factor, is the plant genotype (Fig. 1A). In previous work using legumes and non-legume plants grown in soils from the United Kingdom, we have also observed fraction, followed by soil and plant species to be the main factors (31). Similar importance of fractions, soil type, developmental stage, and genotype was also observed for rice grown in the United States (36, 37). This indicates that the plant presence itself influences the surrounding microbiota, while its exact profile is influenced by other factors, such as soil type.

We can also conclude that soil type is more important for loose soil and rhizosphere fractions, while plant fractions such as rhizoplane and root endosphere are greatly affected by the plant developmental stage and genotype (Fig. S1B). All our statistical analyses indicate that the plants exert a gradient of influence, which is greatest toward the root and decreasing toward surrounding soil (Fig. 1B).

Irrespective of a sample’s geographical origin (India or United Kingdom), plant species, or soil type, the rhizoplane and root endosphere are colonized by *Alphaproteobacteria* and *Bacteroidetes* (Fig. 2A, Fig. S4 and S6). *Proteobacteria*, and especially their alpha class are common root colonizers found across multiple soils and plant species, such as *Arabidopsis* (33), *Lotus* (14), barley (38), and rice (36). The class *Alphaproteobacteria* harbors bacterial taxa that are likely to be quick in metabolizing plant-derived nutrients (39), and many of them may have genomic traits similar to those of plant symbionts (40).

*Bacteroidetes* along with *Proteobacteria*, *Actinobacteria*, and *Firmicutes* were found to contain many genetic adaptations to interact with plant hosts (41). However, their abundance is also correlated with pathogen presence. *Cytophaga* spp. and *Flavobacterium* spp. reduce pathogen load inside the infected plant roots by antibiotic production (35). While we have not tested any *Bacteroidetes* isolates for antifungal properties, we found three pigeon pea varieties to have an increased abundance of these genera in various soil types.

The main root-inhabiting genera were *Rhizobium*, *Pseudoxanthomonas*, and *Sphingopyxis* for vegetative-stage plants, suggesting that these genera are especially attracted by young plants, possibly by an active plant secretion. Some plant genotypes, when grown in a specific soil, also had a high abundance of *Bradyrhizobium*, suggesting an active endosymbiosis. Vegetative plants were associated with *Sphingopyxis* root colonization, which was replaced by *Brachymonas* in the flowering stage (Fig. 2C).

The *Sphingopyxis* and *Brachymonas* genera are rarely found in plant microbiomes. A *Sphingopyxis* isolate was found to be an inconsistent root colonizer in competition with a synthetic community (42). However, in our case, this genus was consistently associated with roots, irrespective of the soil type or plant genotype. Conversely, we consider *Brachymonas* to be an opportunistic root colonizer of older plants that no longer invest resources in interaction with their microbiota (43). In general, young plants strongly associate with only a part of the surrounding microbiota, as the bacterial diversity is lower on and inside the root than the surrounding soil. However, over time with the flowering stage, the plant loses its selective pressure, allowing various other bacteria to colonize the roots (Fig. 1D).

We confirmed an elevated *Bradyrhizobium* presence for selected genotypes grown in selected soil types (i.e., Asha in inceptisol and Durga in vertisol [Fig. 3B and C]); however, contrary to our expectations, *Bradyrhizobium*’s presence was generally low in the root fraction, while it can be a dominant genus in the surrounding soil. This genus contains free-living, non-symbiotic strains, which can dominate the soil *Bradyrhizobium* community in forest soils (44) and was also found in agricultural soils (45). Here, we have not established what proportion of soil *Bradyrhizobium* contain symbiotic properties, a question worth investigating in the future.

*Rhizobium* was the most abundant root-colonizing species in our 16S rRNA gene amplicon assay (Fig. 3A), and in order to validate the bioinformatics-based conclusion, we used plant inoculation experiments using selected Indian *Rhizobium* and *Bradyrhizobium* strains. We confirmed its dominance over *Bradyrhizobium* with isolation studies from native Indian soils (Fig. 2F). While these *Rhizobium* isolates lack *nod* and *nif* genes and are unable to nodulate pigeon pea, they can outcompete *Bradyrhizobium* in native soils and in gnotobiotic conditions. A similar effect of *Bradyrhizobium* being outcompeted was observed for soybean seedlings containing natural seed epiphytes (46).

Moreover, the case of pigeon pea is not alone, as roots of soybean, the *Bradyrhizobium* host plant, were found to be colonized with a bacterial community where this symbiont contributes to only ∼1% of the population (9, 47). Such low abundance is in contrast with pea plants with ∼10 to 20%, *Medicago* with ∼10 to 60%, or *Lotus* with ∼10% root presence of their respective symbiotic genus, i.e., *Rhizobium*, *Ensifer*, or *Mesorhizobium*, respectively (14, 31, 48–50).

*Bradyrhizobium*, while being abundant in Indian soils, is either a poor root colonizer of its host plant under competition from other soil-dwelling bacteria or, within this species, there are many non-symbiotic strains. Despite this, plants can still be nodulated. *Bradyrhizobium* as a pigeon pea endosymbiont has evolved to be recognized by this legume, infect its root, and develop root nodules. We speculate that symbiotic *Bradyrhizobium* colonizes the emerging root hairs directly from the soil, where its number is high, rather than actively colonizing the root and moving toward the emerging nodule regions. Hence, in this symbiotic partnership, it is not essential to colonize roots in order to form nodules. Pigeon pea is grown in dry conditions so that, as a plant, it can tolerate prolonged droughts, although, an effect of this is to decrease symbiosis efficiency. This feature may explain the poor nodulation of pigeon pea in native soils. Hence, any selection for pigeon pea inoculants should be based not only on their N_2_-fixing potential, but also on their soil endurance and competitiveness. The selected elite strains should be field-tested under different climate conditions and plant varieties to define the best soil-host-symbiont association for the agroclimatic conditions in India.

Further steps in the United Kingdom-India N_2_ fixation research program are to add selected (best N_2_ fixers and well-adapted) *Bradyrhizobia* (identified in our subsequent manuscript) to pigeon pea seed coatings using appropriate inoculant technology to be tested by the participating labs in India.

In this way, we will be able to track if the plants that failed to be nodulated have a substantial *Rhizobium* population around their roots, as that would suggest outcompetition of *Bradyrhizobium* inoculant. Results from such experiments would be able to validate (or not) our main bioinformatic conclusions; however, that is future work.

Moreover, an additional potential solution to low symbiotic efficiency is to develop synthetic communities with *Bradyrhizobium*, *Rhizobium*, and other strains, looking for non-rhizobial species that can support *Bradyrhizobium* and/or reduce *Rhizobium* competitiveness. Such non-rhizobia could be added as a part of the seed coating. For example, strains of *Bacillus* were reported to support *Bradyrhizobium* numbers and *Ensifer* nodulation of soybean in saline soils (51).

## MATERIALS AND METHODS

### Experimental design.

We collected three different soil types from farmers’ fields in the principal pigeon pea producing regions of India during the presowing season between June and August 2017. Alfisols were collected from Andhra Pradesh (Rompicharla, Guntur district; 16.213900°N, 79.921386°E), vertisols from Madhya Pradesh (Athner, Betul district; 21.6406552°N, 77.91300°E), and inceptisols from Uttar Pradesh (Sitamarhi, Allahabad district; 25.2782289°N, 82.28691°E) (Fig. S9A). Rompicharla has a tropical climate with an average annual temperature of 28.5°C (24.1 to 33.6°C) and average annual precipitation (rainfall) of 906 mm. Athner also has a tropical climate, with an average annual temperature of 24.6°C (19.1 to 32.4°C) and average annual precipitation (rainfall) of 943 mm. Sitamarhi has a subtropical climate with an average annual temperature of 25.7°C (16.1 to 34.2°C) and average annual precipitation (rainfall) of 981 mm (Fig. S9B).

10.1128/mBio.00423-21.9 FIG S9 (A, left) Map of India showing GPS locations of pigeon pea sampling sites at Rompicharla (Andhra Pradesh), Athner (Madhya Pradesh), and Sitamarhi (Uttar Pradesh). Map drawn with ArcGIS software. (Right) Photographs depicting the texture and size of seeds of the pigeon pea cultivars Asha (ICPL 87119), Mannem Konda Kandi (MKK) (ICPH 2740), and Durga (ICPL 84031). (B) Soil types used in the study. (Top panel, left to right) inceptisol, vertisol, and alfisol samples for soil physicochemical characterization. (Bottom panel, left to right) Pigeon pea fields in Mirzapur (Uttar Pradesh), Betul (Madhya Pradesh), and Rompicharla (Andhra Pradesh), having inceptisol, vertisol, and alfisol, respectively. Soil samples were collected from the fields pictured above. Download FIG S9, PDF file, 1.4 MB. Copyright © 2021 Chalasani et al. 2021 Chalasani et al. https://creativecommons.org/licenses/by/4.0/ This content is distributed under the terms of the Creative Commons Attribution 4.0 International license.

Physicochemical characterization of the collected soils (Table S1) was performed using HiMedia soil testing kits (HiMedia Laboratories, Mumbai, India) according to the manufacturer’s instructions. Three popular pigeon pea cultivars (genotypes) were selected for this study, viz. Asha (ICPL-87119), Durga (ICPL-84031), and Mannem Konda Kandi (MKK; ICPH-2740) (Table S1). The seeds were procured from the International Crops Research Institute for the Semi-Arid Tropics (ICRISAT), Hyderabad, India. Seeds were surface-sterilized using HgCl_2_ (0.1%) and ethanol (70%) and germinated on MS (Murashige and Skoog) agar. Three seedlings of each genotype were transplanted into pots (pot size = 7.5 kg) individually filled with three collected soil samples. The plants were grown using six biological replicates in a glasshouse at the University of Hyderabad, Hyderabad, India, under identical conditions of light, temperature, and humidity until flowering stage. Six pots of soil for each soil type (without growing any plant) were used as the bulk soil control. Plants, as well as control pots, were watered as needed with sterilized distilled water every alternate day without adding any other fertilizers.

### Sampling of soil and root fractions.

Plants were harvested at two developmental stages—vegetative (1 month after seedling emergence) and flowering stages (3 months after seedling emergence for Durga; 4 months after seedling emergence for Asha and MKK). Uprooted plant roots were briefly shaken to remove loosely attached soil, which was collected as “‘loose soil” fraction. The soil bound tightly to the roots was collected without damaging the root and root nodules by vortexing and centrifugation at 4,000 rpm for 10 min to yield the “rhizosphere” fraction. After removing the rhizosphere soil, roots were washed and transferred to 15-ml Falcon tubes (with 10 ml sterile water) and sonicated for 5 min at full intensity in an ultrasonic bath. Roots were removed, and the Falcon tube was centrifuged at 4,000 rpm for 10 min to collect “rhizoplane” fraction as a pellet. Washed and sonicated roots were ground to a powder using liquid nitrogen and defined as the “endosphere” fraction.

### DNA extraction, PCR, and sequencing.

DNA for the 16S rRNA gene amplicon study was extracted from the bulk and loose soil, rhizosphere, rhizoplane, and root endosphere samples (0.3 g each) using a NucleoSpin soil kit (Machery Nagel, Germany) according to the manufacturer’s instructions. The V4 hypervariable region of the bacterial 16S rRNA gene was amplified using double-barcoded 515F/806R primer pairs (52). The PCR mixture consisted of Phusion high-fidelity (0.2 μl), high-fidelity buffer (4 μl) (Thermo Scientific, Waltham, USA), dinucleotide triphosphates (0.4 μl), primers (1 μl of each 10-pmol stock), template DNA (1.5 μl of 5 ng μl^−1^), and H_2_O up to a 20 μl final volume. For rhizoplane and endosphere fractions, peptide nucleic acid (PNA) for targeting plastid (pPNA, 5′-GGCTCAACCCTGGACAG-3′) and mitochondrial (mPNA, 5′-GGCAAGTGTTCTTCGGA-3′) DNA (PNA Bio, Newbury Park, CA, USA) of 1 μM as PCR clamps (53) were added. PCR cycles were as follows: 98°C for 1 min, 35 cycles of 98°C for 30 s, 57°C for 30 s, and 72°C for 45 s, with a final elongation step of 72°C for 7 min. Each DNA sample was amplified in triplicate, followed by purification using a PCR cleanup kit (D4014; Zymo Research). For each amplification run in 96-well plates, water was used as a negative control (no-DNA control). Samples were pooled and sequenced using the Illumina MiSeq platform using v3 chemistry of 300PE run at the Molecular Research DNA laboratory in Texas, USA.

**Processing of sequencing data.** Initial quality filtering and read alignment was done using Usearch 10 fastq_mergepairs with fastq_maxee using an EE score of 1. After barcode removal, only reads of the desired length of 292 bp were used for further analysis. Reads were filtered from plant chloroplast and mitochondria (around 2% of the initial reads were plant origin) using a custom-made Bash script (Table S1). Reads were binned into zero-radius operational taxonomic units (zOTUs), including chimera removal according to the Usearch10 pipeline with Unoise3 (54) and annotated using the SILVA SSU132 16S rRNA database (55).

### Statistical analyses.

Permutational multivariate analysis of variance (PERMANOVA), unconstrained principal-coordinate analysis (PCoA), and analysis of similarity (ANOSIM) were based on Bray-Curtis dissimilarity matrices calculated from standardized, square-root transformed abundance data and calculated and/or visualized in Primer 6 software (PRIMER-E; Quest Research Ltd., Auckland, New Zealand). Factors influencing the bacterial community were statistically assessed using permutation of residuals under a reduced model, sum of squares type III (partial) with 9,999 permutations using an unrestricted permutation of raw data model of PERMANOVA. We considered pseudo-*F* values as proxies of a given factor’s importance for sample separation based on the ratio of the beta-diversity (variation between two or more sample groups) to alpha-diversity (variation between individual samples inside each of these groups). PCoA plots are designed to visualize distance matrices with maximum sample separation along multiple axes (however, for clarity only the first two axes are shown) without prior factorial description. One-way ANOSIM tests based upon 9,999 permutations were used to calculate the difference (the ratio of beta- to alpha-diversity) between each set of data for a given factor. Similarity percentage (SIMPER) was also run in PRIMER 6 on standardized and square-root transformed abundance data and aimed to identify the bacterial community taxa with the greatest influence on sample group separation.

The Shannon entropy plot, Volcano plot, and taxonomic bar plots were visualized in PRISM 8 (GraphPad, San Diego, USA). Shannon entropy was calculated for each sample as (–1) · sum of (each zOTU value · ln of each zOTU value), where the sum of zOTU values for each sample equals 1. For Volcano and taxonomic plots, the taxonomic affiliations of zOTUs were summed into phyla, families, and genera. Volcano plots present genera locations on an XY matrix as a result of their fold change against bulk soil (*x* axis) and statistical significance of this change (*y* axis) corrected for multiple testing with false-discovery rate (FDR).

### Isolation of bacteria from the rhizosphere, roots, and nodules.

Pigeon pea plants (three cultivars each in three soil types) were harvested at the vegetative stage for isolation of bacteria. Harvested nodules were surface-sterilized with HgCl_2_ (0.1%), crushed, serially diluted in saline (0.86% NaCl), streaked onto CRYEM (Congo red yeast extract mannitol) plates, and incubated at 25°C for up to 7 days. Colonies were selected from CRYEM plates and streaked onto new plates to obtain pure cultures. Rhizosphere and root samples were collected as described above, diluted in saline, streaked onto yeast extract mannitol (YEM) plates, and incubated at 30°C for up to 4 days. Single colonies were purified further, their BOX-PCR was obtained using the BOX-A1R primer 5′-CTACGGCAAGGCGACGCTGACG-3′ (56), and their 16S rRNA gene was sequenced after PCR amplification using the 27F (5′-GTTTGATCCTGGCTCAG-3′) and 1494R (5′-ACGGCTACCTTGTTACGACTT-3′) primers.

### Whole-genome sequencing (WGS) of pigeon pea isolates.

Eighteen pigeon pea isolates were selected based on their different BOX-PCR pattern. Culture samples were provided to Microbes NG for Illumina sequencing (MiSeq v2; paired ends [PE], 2 × 250 bp). The closest available reference genome for each sample was identified with Kraken v2 (57), and reads were mapped to this using the Burrows-Wheeler Transform MEM algorithm (BWA-MEM) v07.17 (58) to assess the quality of the data. *De novo* assembly was performed with SPAdes v3.14.1 (59). An automated annotation was performed using Prokka v1.12 (60). Strains were annotated using their whole genomes with EzBioCloud to the species level.

A local BLAST database of these genomes was generated in Geneious R10 v10.2.6, and *nodC* and *nifH* sequences from Bradyrhizobium cajani AMNPC 1010^T^ (BioProject number PRJNA593773) and Ensifer fredii NBRC 14780^T^ (BioProject number PRJDB6002) were used to assess the presence of these genes in bacteria belonging to *Burkholderia/Paraburkholderia* spp. (4 isolates), *Microbacterium* spp. (3 isolates), and *Rhizobium/Agrobacterium* spp. (11 isolates).

### Gnotobiotic inoculation assay.

Seeds were surfaced-sterilized using ethanol (70%) for 1 min and bleach (4%) for 3 min and placed on water agar until the seedling emergence. Plants were moved to pots (1 liter) with vermiculite with N-free rooting solution (400 ml) (61) in a controlled growth chamber and inoculated either with a single *Rhizobium* or *Bradyrhizobium* strain, a community of *Rhizobium* spp., or a community of *Bradyrhizobium* spp. or were double-inoculated with both the *Rhizobium* spp. and *Bradyrhizobium* spp. communities. *Rhizobium* strains were isolated from the roots, while *Bradyrhizobium* strains came from pigeon pea nodules. *Rhizobium* strains come from this work, while *Bradyrhizobium* strains were isolated from pigeon pea nodules grown in various Indian soils. Characterization of *Bradyrhizobium* strains will be covered in a separate publication.

The 4-week-old pigeon pea plants were harvested, their roots (rhizoplane and endosphere combined) and nodules (if any) were crushed using a pestle and mortar, and the crushed nodule macerate was plated in dilution series on AG (arabinose-gluconate) agar plates and left for 3 days to allow the growth of both *Rhizobium* and *Bradyrhizobium* spp. DNA from up to 5 individual colonies from highly diluted treatments was isolated and used for ribosomal intergenic spacer (RISA) fingerprinting for species identification using RISA primers as follows: ITSF (5′-GTCGTAACAAGGTAGCCGTA-3′) and ITSReub (5′-GCCAAGGCATCCACC-3′) and PCR conditions of 95°C for 7 min, 30 cycles of 95°C for 30 s, 55°C for 30 s, 72°C for 1 min, and final elongation of 72°C for 7 min. All *Bradyrhizobium* strains used have a RISA band of ∼900 bp, while all *Rhizobium* strains have a RISA band of approximately 1,200 bp, allowing for quick species identification.

### Availability of data and materials.

16S rRNA gene sequencing data and associated metadata were deposited to the EMBL-EBI SRA repository under accession code PRJEB39218. The genome data are stored in the GenBank database as BioProject PRJNA693523. Detailed documentation of the bioinformatic pipeline and data analysis output used for figure preparation and statistical analysis can be found in Table S1.
