# Supplementary figures and images for: Poor Competitiveness of Bradyrhizobium in Pigeon Pea Root Colonization in Indian Soils
Source: mBio. 2021 Jul 6;12(4):e00423-21. doi: 10.1128/mBio.00423-21 (PMC8406239; doi:10.1128/mBio.00423-21)

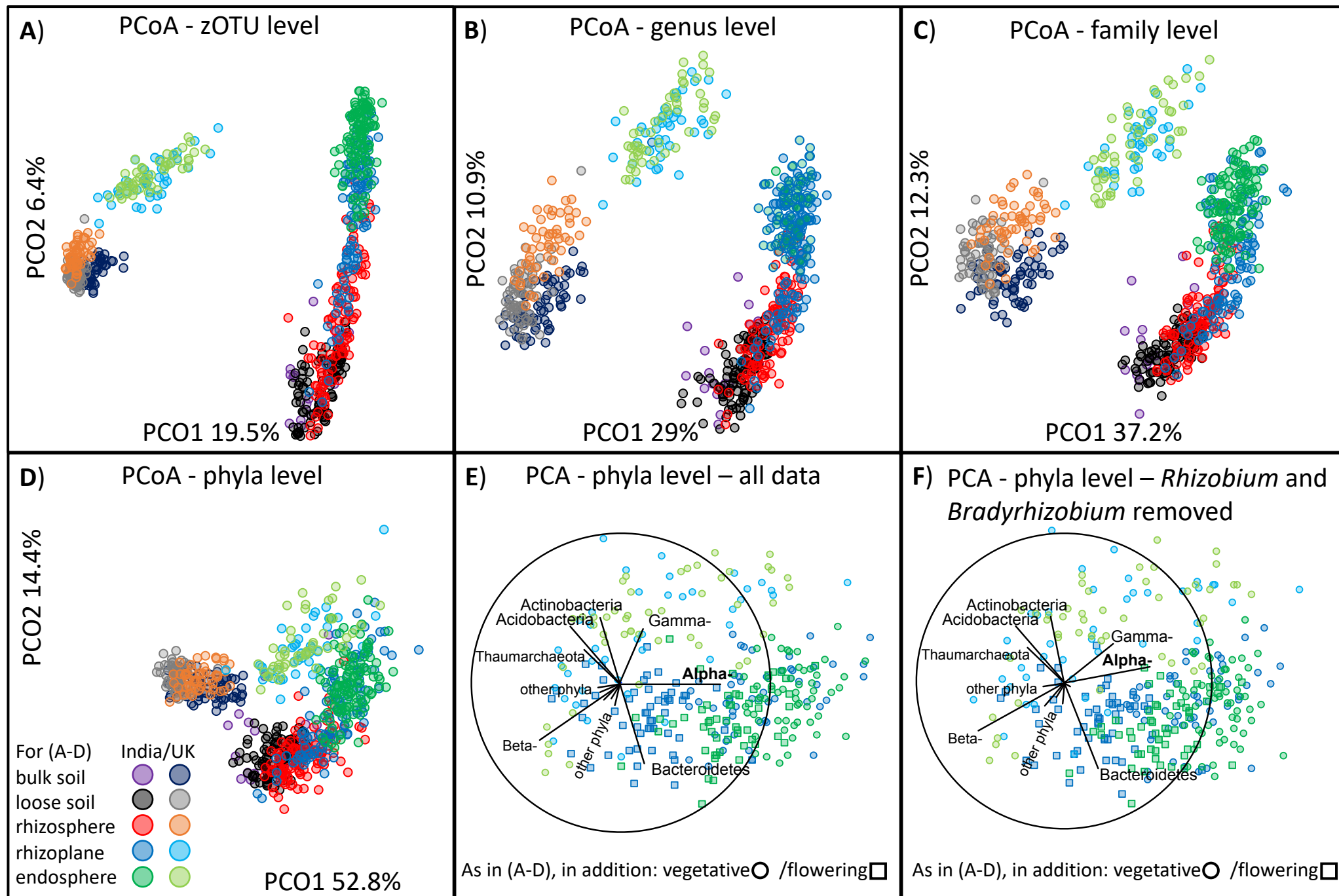

Supplement: FIG S4 [file mbio.00423-21-sf004.pdf]

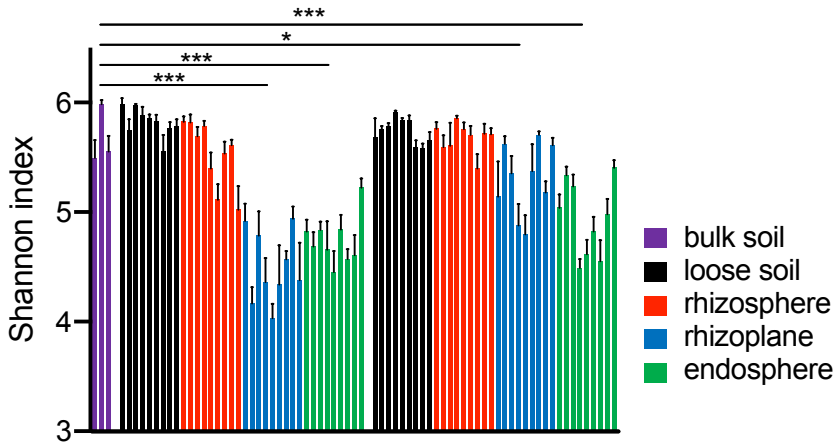

Supplement: FIG S5 [file mbio.00423-21-sf005.pdf]

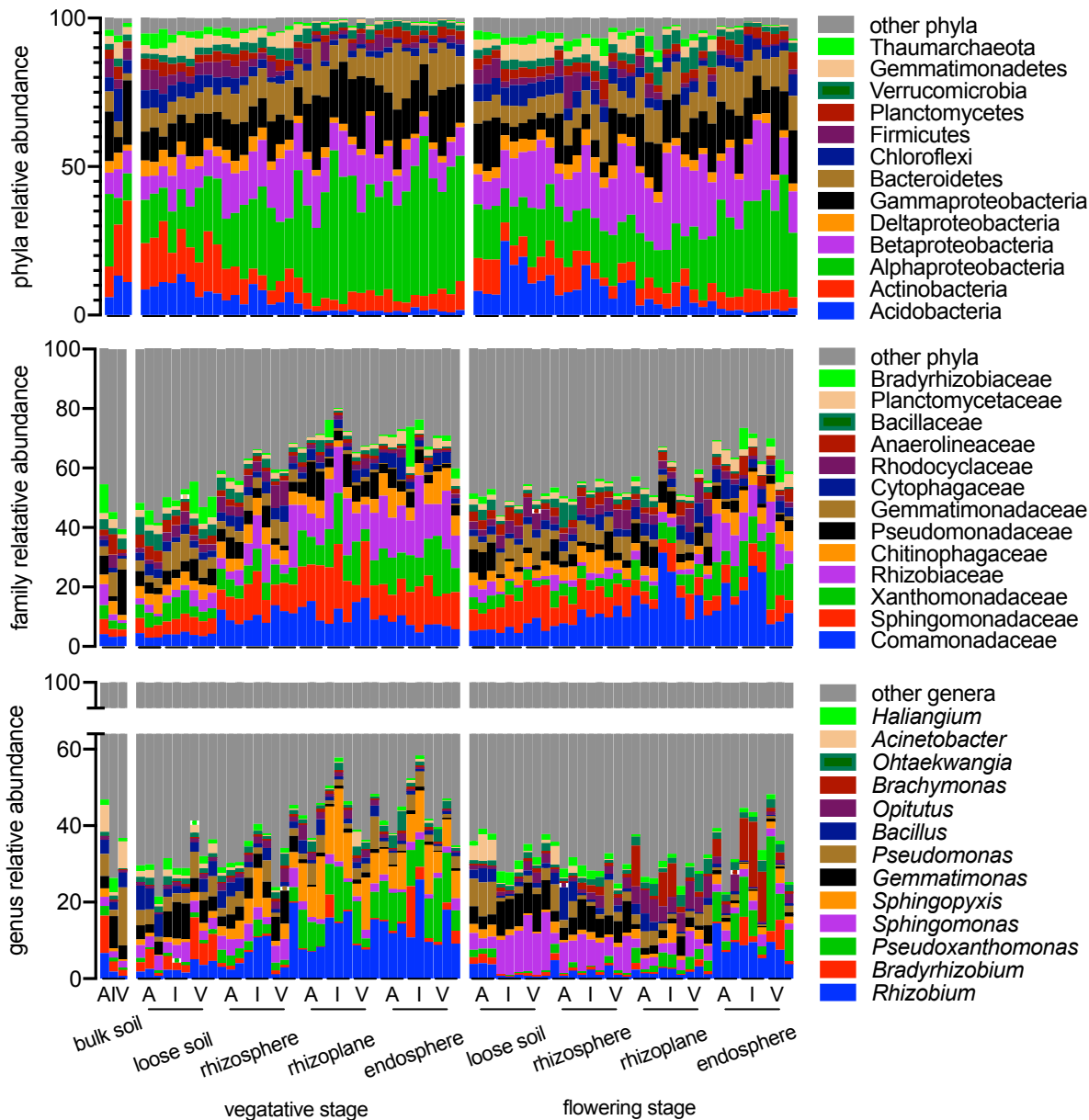

Supplement: FIG S6 [file mbio.00423-21-sf006.pdf]

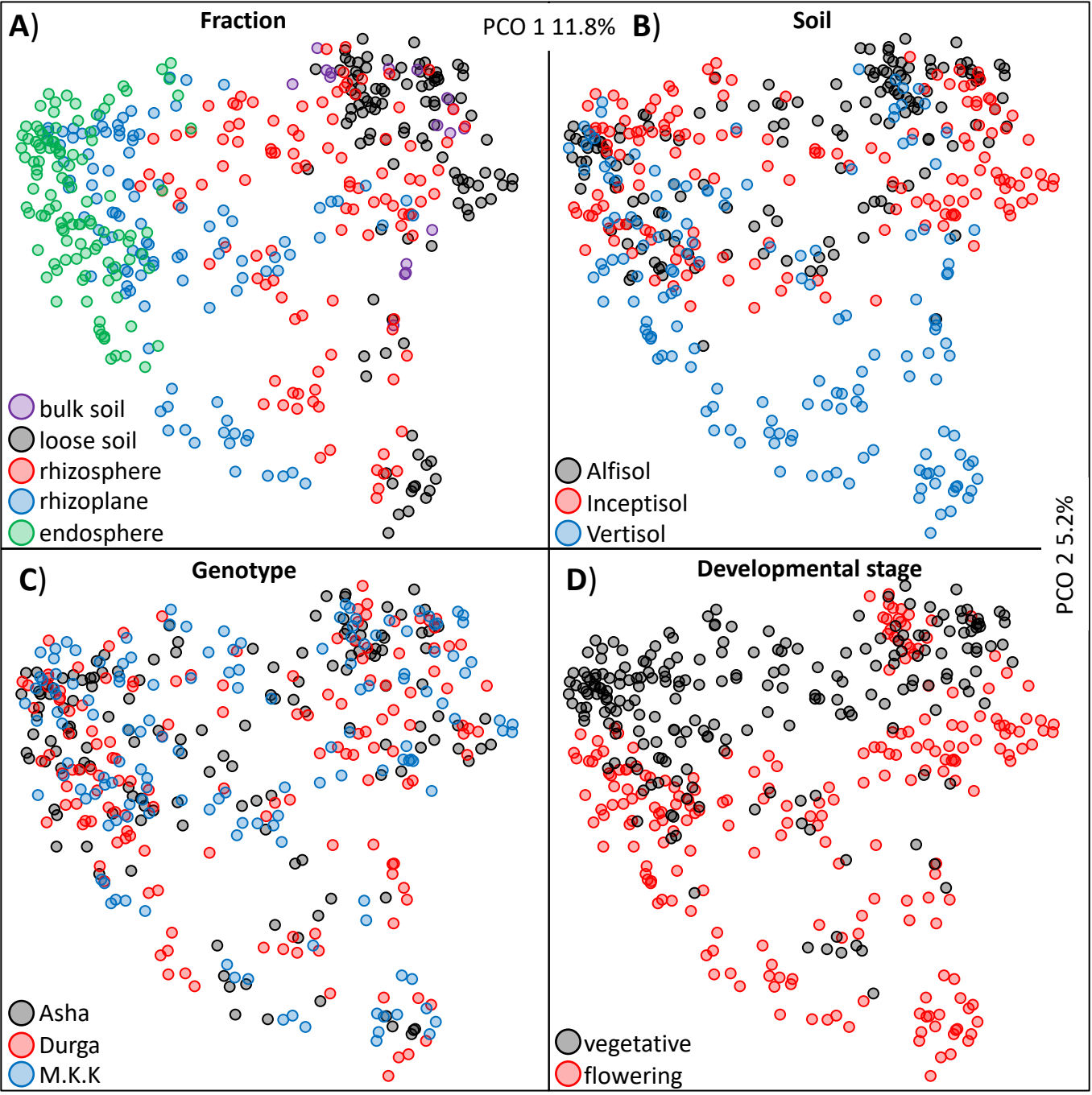

Supplement: FIG S2 [file mbio.00423-21-sf002.pdf]

phyla level

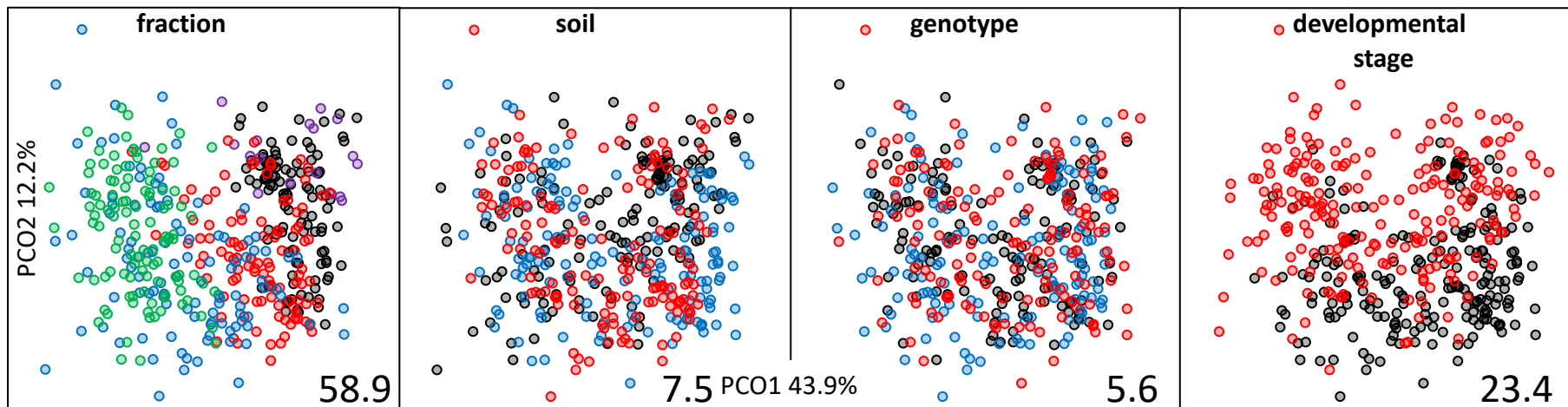

family level

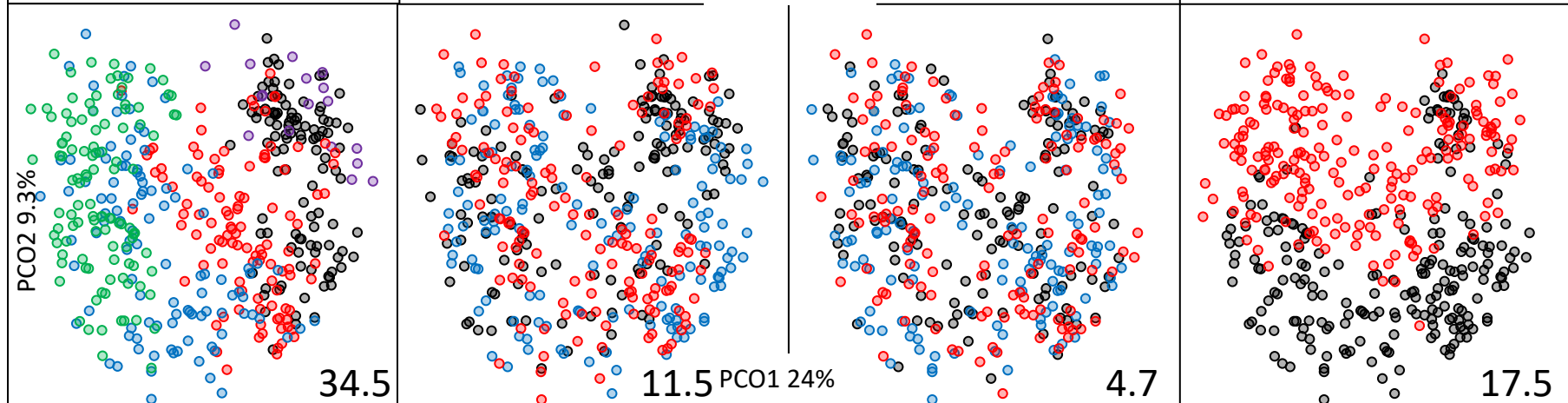

genus level

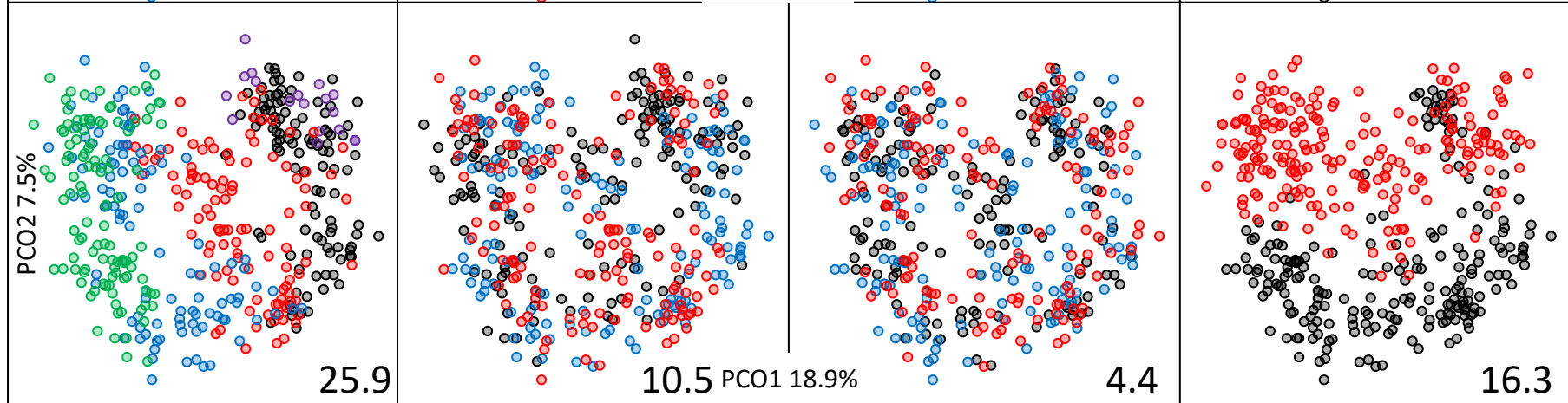

Supplement: FIG S3 [file mbio.00423-21-sf003.pdf]

A)

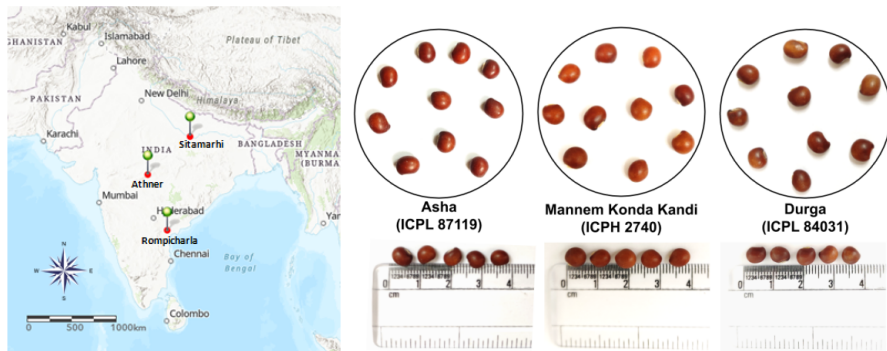

B)

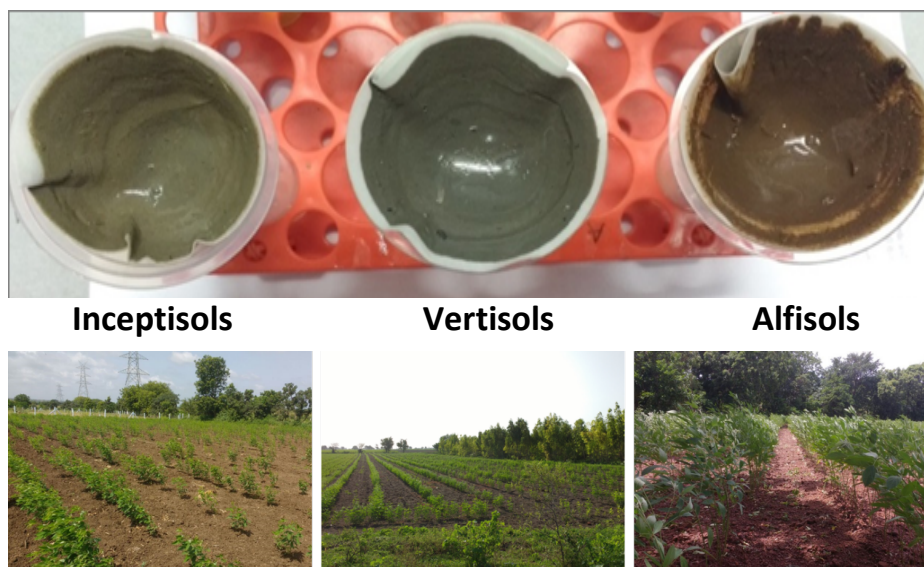

Supplement: FIG S9 [file mbio.00423-21-sf009.pdf]
