# Supplementary material for: Poor Competitiveness of Bradyrhizobium in Pigeon Pea Root Colonization in Indian Soils
Source: mBio. 2021 Jul 6;12(4):e00423-21. doi: 10.1128/mBio.00423-21 (PMC8406239; doi:10.1128/mBio.00423-21)
Supplement: FIG S7 [file mbio.00423-21-sf007.pdf]

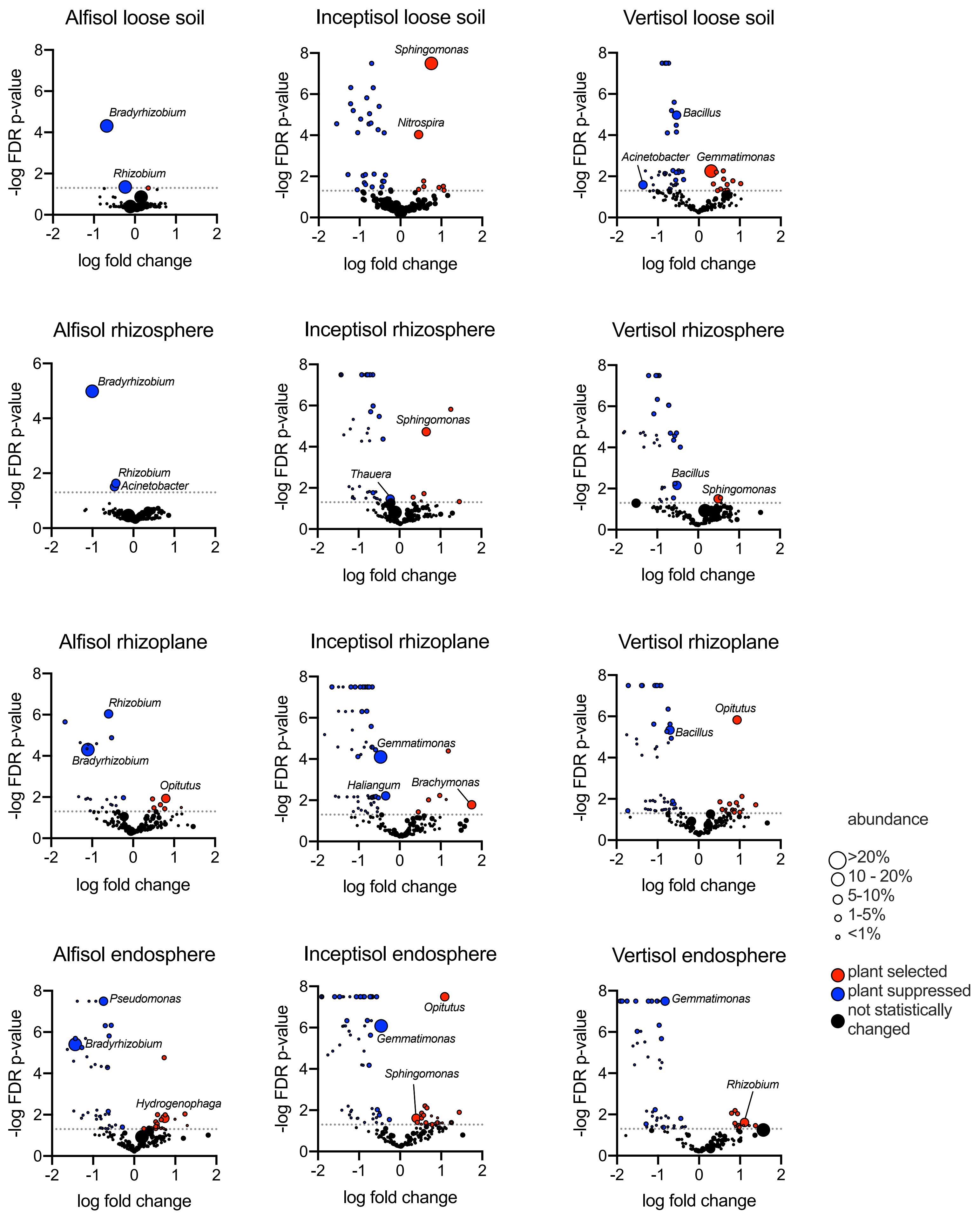

Alfisol loose soil
Bradyrhizobium
Rhizobium
Inceptisol loose soil
Sphingomonas
Nitrospira
Vertisol loose soil
Bacillus
Acinetobacter
Gemmatimonas
Alfisol rhizosphere
Bradyrhizobium
Rhizobium
Acinetobacter
Inceptisol rhizosphere
Sphingomonas
Thauera
Vertisol rhizosphere
Bacillus
Sphingomonas
Alfisol rhizoplane
Rhizobium
Bradyrhizobium
Opitutus
Inceptisol rhizoplane
Gemmatimonas
Haliangum
Brachymonas
Vertisol rhizoplane
Opitutus
Bacillus
Alfisol endosphere
Pseudomonas
Bradyrhizobium
Hydrogenophaga
Inceptisol endosphere
Opitutus
Gemmatimonas
Sphingomonas
Vertisol endosphere
Gemmatimonas
Rhizobium
-log FDR p-value
log fold change
abundance
>20%
10 - 20%
5-10%
1-5%
<1%
plant selected
plant suppressed
not statistically changed
