# Supplementary material for: Poor Competitiveness of Bradyrhizobium in Pigeon Pea Root Colonization in Indian Soils
Source: mBio. 2021 Jul 6;12(4):e00423-21. doi: 10.1128/mBio.00423-21 (PMC8406239; doi:10.1128/mBio.00423-21)
Supplement: FIG S8 [file mbio.00423-21-sf008.pdf]

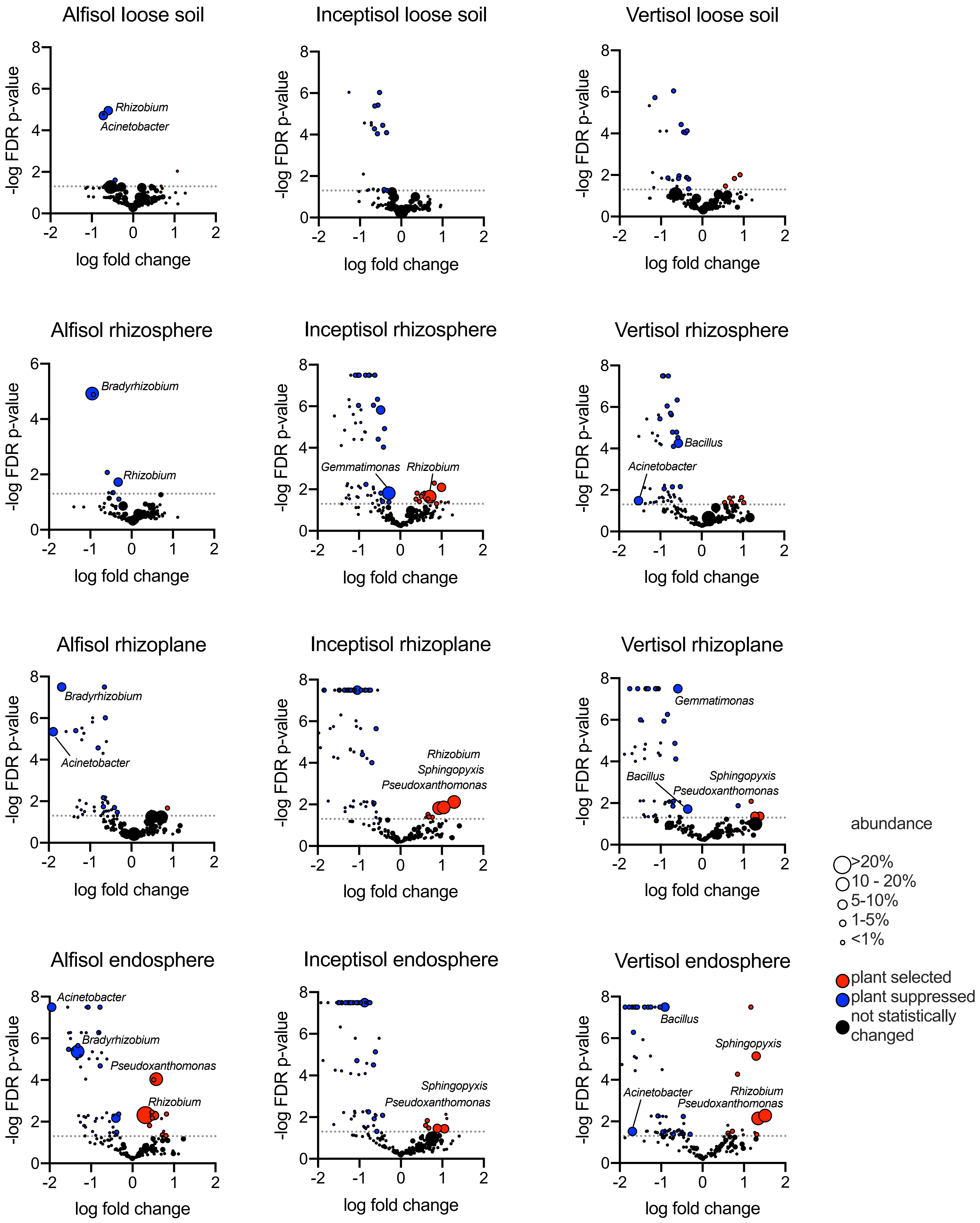

Alfisol loose soil
Inceptisol loose soil
Vertisol loose soil
Rhizobium
Acinetobacter
Alfisol rhizosphere
Inceptisol rhizosphere
Vertisol rhizosphere
Bradyrhizobium
Rhizobium
Gemmatimonas
Rhizobium
Bacillus
Acinetobacter
Alfisol rhizoplane
Inceptisol rhizoplane
Vertisol rhizoplane
Bradyrhizobium
Acinetobacter
Rhizobium
Sphingopyxis
Pseudoxanthomonas
Gemmatimonas
Bacillus
Sphingopyxis
Pseudoxanthomonas
Alfisol endosphere
Inceptisol endosphere
Vertisol endosphere
Acinetobacter
Bradyrhizobium
Pseudoxanthomonas
Rhizobium
Sphingopyxis
Pseudoxanthomonas
Bacillus
Sphingopyxis
Acinetobacter
Rhizobium
Pseudoxanthomonas
-log FDR p-value
log fold change
abundance
>20%
10 - 20%
5-10%
1-5%
<1%
plant selected
plant suppressed
not statistically changed
