# Supplementary material for: Poor Competitiveness of Bradyrhizobium in Pigeon Pea Root Colonization in Indian Soils
Source: mBio. 2021 Jul 6;12(4):e00423-21. doi: 10.1128/mBio.00423-21 (PMC8406239; doi:10.1128/mBio.00423-21)
Supplement: FIG S1 [file mbio.00423-21-sf001.pdf]

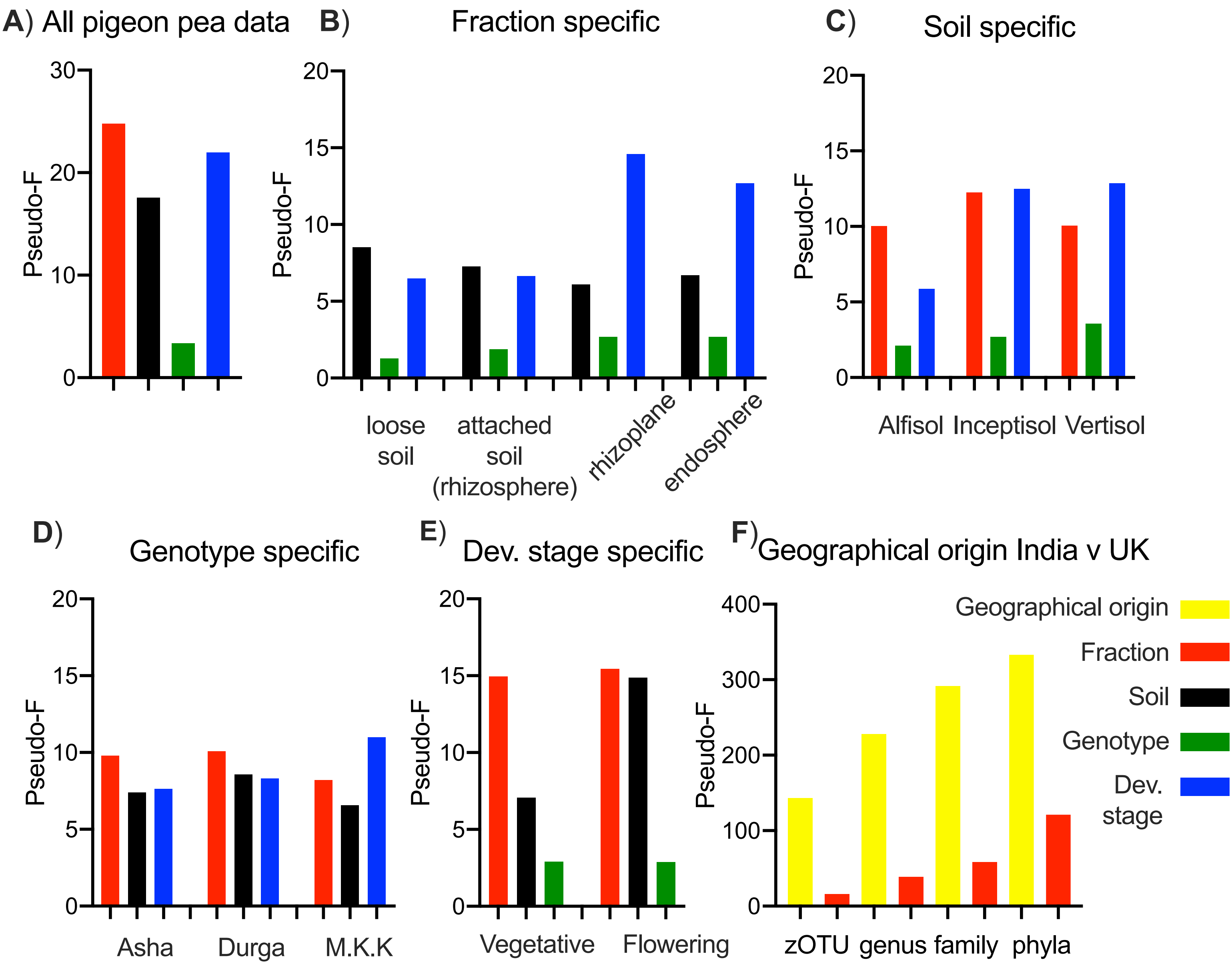
A) All pigeon pea data
B) Fraction specific
C) Soil specific
Pseudo-F
30
20
15
10
5
0
loose soil
attached soil (rhizosphere)
rhizoplane
endosphere
Alfisol
Inceptisol
Vertisol
D) Genotype specific
E) Dev. stage specific
F) Geographical origin India v UK
Asha
Durga
M.K.K
Vegetative
Flowering
400
300
200
100
zOTU
genus
family
phyla
Geographical origin
Fraction
Soil
Genotype
Dev. stage
